# An Alternative Technique for Topical Application of Acidulated Phosphate Fluoride (APF) Gel: A Two-Years Double-Blind Randomization Clinical Trial (RCT)

**DOI:** 10.3390/medicina59122118

**Published:** 2023-12-04

**Authors:** Ana Laura Sorazabal, Pablo Salgado, Silvana Ferrarini, Rocio Lazzati, Aldo Fabian Squassi, Guglielmo Campus, Graciela Klemonskis

**Affiliations:** 1 Cátedra de Odontología Preventiva y Comunitaria, Facultad de Odontología, Universidad de Buenos Aires, Buenos Aires C1122AAH, Argentina; ana.sorazabal@odontologia.uba.ar (A.L.S.); pablo.salgado@odontologia.uba.ar (P.S.); silvana.ferrarini@odontologia.uba.ar (S.F.); rocio.lazzati@odontologia.uba.ar (R.L.); aldo.squassi@odontologia.uba.ar (A.F.S.); graciela.klemonskis@odontologia.uba.ar (G.K.); 2 Instituto de Investigaciones en Salud Pública, Facultad de Odontología, Universidad de Buenos Aires, Buenos Aires C1122AAH, Argentina; 3 Department of Restorative, Preventive and Pediatric Dentistry, University of Bern, Freiburgstrasse 7, 3012 Bern, Switzerland; 4 Department of Cariology, Saveetha Dental College and Hospitals, SIMATS, Poonamallee High Road, Chennai 600077, India

**Keywords:** fluoride application, caries management, caries treatment needs index, RCT

## Abstract

*Background and Objectives:* Dental caries is still a dramatic public health problem. The effectiveness of an alternative technique using acidulated phosphate fluoride (APF) gel pH 3.5 was evaluated and compared with conventional treatments in reducing dental caries incidence. *Materials and Methods:* A double-blind randomized controlled trial was conducted, involving 180 children aged 4–11 years. Three groups were formed: NaF varnish (NaFV), APF in tray (APFt), and APF in toothbrush (APFtbru). Clinical examinations were performed using standardized criteria and the ICDAS Index was assessed. The Caries Treatment Needs Index (CTNI) was calculated. Dental examinations were conducted at baseline, 12 months, and 24 months. *Results*: At baseline, 16,719 dental surfaces were included, with 15,434 surfaces being sound. After 24 months, the Kaplan–Meier analysis showed significant differences between the APFtbru group and the other two groups (*p* = 0.03). Cox regression analysis revealed that the surfaces treated with NaF varnish had the lowest survival rates (HR = 0.51 95%CI = 0.33/0.81). Occlusal surfaces had the lowest cumulative survival, while other tooth surfaces showed similar performance. *Conclusions*: The alternative technique of APF gel application with a toothbrush resulted in maintaining dental health over 24 months. This technique can be cost-effective and may offer advantages over traditional fluoride varnish application.

## 1. Introduction

Dental caries aetiology involves a complex interaction between tooth structure, host, microbial biofilm, and sugar exposure, and still a large number of lesions are not treated [1,2].

Although caries prevalence figures dropped after the increased availability of fluoridated toothpastes, oral conditions, and particularly dental caries and its consequences, have major public health impacts for the entire global population [3,4] underlying the importance of educating the population about the environmental impact of caries prevention programmes and dental treatments as caries may negatively influencing speech, aesthetics, quality of life, the masticatory system, and the dental arches [5,6].

As mentioned above, the main key to preventing caries disease is fluoride, especially its topical use. Topical fluoridated products are able to create reservoirs, providing bioavailability of the product for the dental surface which is effective in reducing demineralization and increasing remineralization [2,3]. Professional fluoride application (PFA) for the prevention and management of dental caries has been used for over 50 years, using products containing high concentrations of fluoride usually formulated in gel, foam, and varnish form.

Among professional fluoride formulations, gels and varnishes stand out as the most effective in caries prevention; however, its anticaries effect highlights the need for economic evaluations [7,8,9,10].

In children, the application of topical fluoride at 3-month intervals is probably the most effective in preventing caries in pre-school children [11]. Several systemic reviews underline the considerable caries-inhibiting effect with moderate certainty of evidence, providing a 37% reduction in decayed, missing, and filled tooth surfaces [11,12,13,14]. However, the evidence was inconclusive.

Caries figures in Argentine children are quite high even if no national data are present [15].

Starting from this premise, the objective of this paper was to evaluate the effectiveness of the use of an alternative technique for the application of acidulated phosphate fluoride (APF) gel pH 3.5 (12,300 ppm) and to compare it with topical conventional treatments in incidence of dental caries.

## 2. Materials and Methods

### 2.1. Trial Design and Participants

This study was designed as a double-blind three-parallel-group randomized controlled trial, in which the subject, operator, and evaluator were masked to the group assignment. It was accepted by the Comisión de Ética, Facultad de Odontología, Universidad de Buenos Aires No. FOUBA 29/05/2013-52.

A sample size calculation was performed using G*Power10 (3.1 https://www.psychologie.hhu.de/arbeitsgruppen/allgemeine-psychologie-und-arbeitspsychologie/gpower, accessed on 12 February 2020) [16], considering the tooth surface as a unit of analysis.

An effect size 0.10 for the new treatment was set with an α err prob = 0.05 and a Power (1-β err prob) = 0.95. The non-centrality parameter λ resulted in 14.18 with a critical χ^2^_(3)_ = 7.81. The total sample size calculated was of 1717 surfaces (20 children) with an overall power of 0.95.

The study was carried out between 2019 and 2022 at two schools presenting homogeneous characteristics in terms of social risk.

The children (*n* = 244) attending those primary schools were the population object of the trial. The randomization process was performed using Excel 2016 using systematic cluster sampling with each school class as a cluster and compiled into a list and three groups were created. The first cluster was randomly chosen, while the others were selected at the systematic interval of three classes. The number of subjects were approximately the same in each class (range 20–24 children).

All parents/caregivers of the children received a leaflet requesting their approval to enrol their children; for those whose families decided not to be included into the trial, the children received the dental treatment needed, but their data were not evaluated. The positive reply rate was 96.7% (*n* = 236).

The inclusion criteria were children between 4 and 11 years old attending the selected schools. Exclusion criteria: children with systemic diseases or systemic disease treatments, motor disorders, and children receiving preventive measures in other public, private, or social-security dental services; one-hundred-eighty subjects were enrolled and three groups were randomly created via a systematic cluster sampling procedure using Excel^®^ software (version 16.79.2) with the criteria of having the same number of subjects in each group and with similar Caries Treatment Needs Index (CTNI) [15] (see Table 1 for a comprehensive description of CTNI).

Each child underwent a clinical examination to determine dental status according to the ICDAS II criteria 14, performed under standardized conditions, using light, dental mirror, WHO probe, and magnification (2.5×). The dental exams were performed by 3 calibrated researchers (S.A., F.S., L.R.). The calibration was carried out prior to the start of the trial with the reference examiner (A.S.).

The calibration strategy consisted of:

(a) Expository class (2 h) with photographs (*n* = 36) aimed at the recognition of the categories established in the ICDAS II14 and the cut-off points between the different categories and the protocol to carry out the diagnosis.
(b) Caries detection using extracted teeth (*n* = 30) (ex vivo 2 h). The examination of the pieces was carried out after drying the surfaces with aerosol compressed air and with adequate lighting. Each operator recorded the observed findings, according to the lesion and activity criteria for each tooth surface. Subsequently, a space for discussion of the results was generated with the benchmark examiner (A.S.).
(c) Clinical practice (20 h), which included the following phases:
  I Assignment to each operator of 6 volunteer subjects who provided a balanced number of dental surfaces with ICDAS14 codes from 1 to 6.
  II Observation and recording of the findings in an ad hoc spreadsheet, in the charge of each independent operator. The visual–tactile clinical examination was performed with frontal light, WHO probes, magnification (3.5×), and drying of surfaces with air.
  III Re-evaluation (one-week after) of each patient by the reference examiner and recording of the findings.

Inter- and intra-examiner reliability was evaluated by comparing the benchmark and the examiners’ outcomes and the percentage of agreement using Cohen’s kappa statistic for sound surfaces, enamel and dentine lesions, and caries activity.

On the basis of the clinical examination, beside the ICDAS14,15 score, the CTNI (11) was also computed. The CTNI13 is based on the interaction of two axes: one based on the progression of the lesion and the other on the technological resources needed to control the risk of dental caries. The progression axis identifies the magnitude of severity and extent. The magnitude of severity component identifies the process of tissue involvement of the dental caries lesion, moving from the white spot lesion to the subsequent progression of the lesion towards cavitation. The magnitude of extension in the “oral unit” is expressed by the number of oral quadrants with visible lesions. The technological axis includes the risk component and the available technological development component. The risk component is the result of the identified variables while the technological development is based on contextualized scientific evidence and is expressed as the appropriate strategies and their application per oral unit and teeth, according to the extent recorded in the dental quadrants.

No instrumentation is required for its determination. It is a visual index whose only requirement is good lighting and a clean mouth.

### 2.2. Intervention

As described above, the children were randomly divided into three different groups according to the mode of treatment:

- NaF varnish group (NaFV) *n* = 60 children received professional application of 5% sodium fluoride varnish twice a year with a pH 7 (ClinPro White Varnish^®^) according to manufacturer’s instructions. (Begin toothbrushing without toothpaste. Open unit-dose package and dispense between 0.25 mL/0.40 mL, mix the varnish, remove excessive saliva by blotting the tooth surfaces with cotton, apply the varnish over all dental surfaces. After application, instruct the patient to close their mouth to set Clinpro varnish. During the following 12 h instruct the patient to avoid hard and sticky foods, tooth brushing, and flossing).
- APF in tray group (APFt) *n* = 60 children received professional application of APF 1.23% gel by tray twice a year. pH 3.5 (Klepp^®^) was applied with tray according to manufacturer’s instructions. (Begin toothbrushing without toothpaste. Remove excessive saliva by blotting the tooth surfaces with cotton, fill applicator tray 1/3 full of gel, insert filled tray into patient’s mouth and instruct to bite down gently for one minute. After treatment time is completed, remove tray, and have patient expectorate residual gel. Instruct patient not to rinse, eat, or drink for 30 min).
- APF in toothbrush group (APFtbru) *n* = 60 children received professional application of APF 1.23% twice a year. pH 3.5 (Klepp^®^) was applied with toothbrush 2 min brushing according to manufacturer’s instructions. (Begin toothbrushing without toothpaste. Spread a 2 cm line of APF gel on a toothbrush. Apply the gel with the toothbrush on all tooth surfaces. Move the brush gently with 2 to 3 anterior–posterior movements in each quadrant of the mouth, starting with the upper right quadrant and repeating in each quadrant following a clockwise direction. Repeat for two minutes. After time is completed, remove toothbrush, and have patient expectorate residual gel. Instruct patient not to rinse, eat, or drink for 30 min).

Three-row children’s brushes with soft, rounded nylon bristles, a compact head and a straight handle were used for all groups.

Enrolment in each group was performed by balancing the CNTI13 categories in each group.

A protocol was applied to all children, based on dental care programs to be carried out in school and dental clinics, with the aim of controlling caries lesions. All the children received teaching and control of personal oral hygiene, dietary counselling, and calculus removal. Caries lesions treatment was divided according to ICDAS code, extension, and severity of the lesions and also according to the dentition. For initial (1–2) inactive caries, active monitoring was done. For initial (1–2) active caries, the care option for primary dentition was silver diamine fluoride (SDF) 38% and for permanent dentition 5% fluoride varnish. For enamel (3) inactive caries, SDF for primary and fluoride varnish for permanent. For m (3) active caries, SDF for primary and permanent molar teeth. For extensive (4–5–6) caries, SDF or atraumatic restorative treatment (ART) were the selected care options for primary teeth according to the extension; meanwhile, for permanent teeth, ART was chosen.

### 2.3. Dental Examination and Monitoring

Observations and clinical examinations were conducted at schools. After 12 and 24 months, the clinical examination was repeated to assess the dental status of schoolchildren following the same criteria described. The presence of new caries lesions was taken as a dependent variable. So, sound surfaces on baseline were observed for 24 months.

In all children, daily brushing was performed with the same toothpaste and toothbrush, supervised by schoolteachers, with fluoride toothpaste (1400 ppm) at school.

### 2.4. Statistical Analysis

All the data were input into a spreadsheet (Microsoft Excel 2021 for Mac, version 16.4.8). A chi-square test was performed for CTNI changes between experimental groups and follow-up examinations; moreover, tests for trends across ordered experimental groups were tested using the Cuzick’s test trends.

Cox Proportional Hazards models were run to assess the factors associated with caries’ change of status. Estimates are reported in the hazard ratio (HR) and their respective 95% confidence interval (95% CI). The Kaplan–Meier estimator was endorsed to estimate the survival fraction of teeth measured as the change of status during the trial. The Efron method was used to handle tied failures. The Greenwood’s formula was used to approximate the variance of the Kaplan–Meier estimator. For all statistical analyses, the statistical significance was set at α = 0.05.

## 3. Results

**Figure 1:**
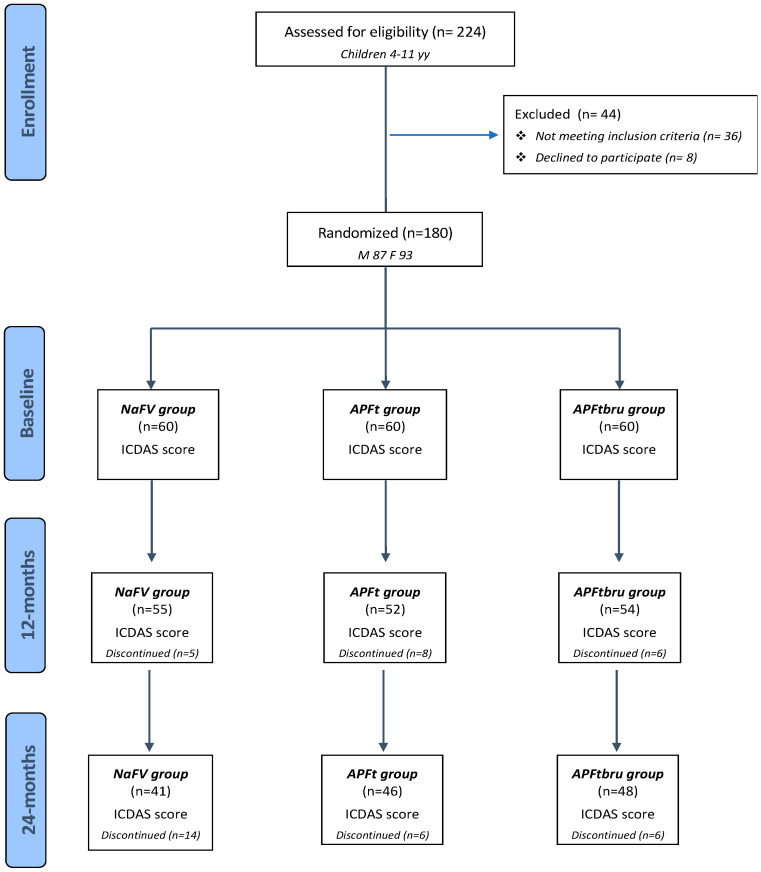
Flow chart.

Overall, 16,719 dental surfaces from 180 kids were included in this study. The CONSORT flow-chart is displayed in Figure 1.

The calibration procedure was successfully performed. The level of agreement overall ranged from 90.44% for caries lesions at enamel level (ICDAS 2) to 94.50% for caries lesions at deep dentinal level (ICDAS 6). The Cohen’s kappa regarding the intra-examiners’ reliability ranged from 0.71 to 0.79.

The distribution of the surfaces by caries status recorded using the ICDAS and the distribution of the subjects by CTNI are displayed in Table 2.

At baseline, the percentage of surfaces with no caries lesions and surfaces affected were in statistically significant association among the three groups (χ^2^_(2)_ = 37.54 *p* < 0.01), while no statistically significant association was observed among the three management groups and CTNI approach (χ^2^_(4)_ = 8.23 *p* = 0.08). Overall, 15,434 surfaces were sound at the baseline examination. During the trial, more than one quarter of the subjects dropped out, the drop-rate was 31.67% (*n* = 19) for group NaF Varnish, 23.33% (*n* = 14) for group APF in tray, and 20.00% (*n* = 12) for group APF in toothbrush: the mean reasons for dropout were the family moving to another area or family/child deciding to leave the trial. So, 135 children (8879 dental surfaces) concluded the experimental period.

The changes of the CTNI approach during the trial in the three experimental management groups (Table 3) were not in statistically significant association within each group, while between experimental groups, the CTNI changes were in statistically significant association at the follow-up examinations (*p* = 0.01 and *p* = 0.02 for the 12th and 24th months evaluation, respectively).

**Figure 2:**
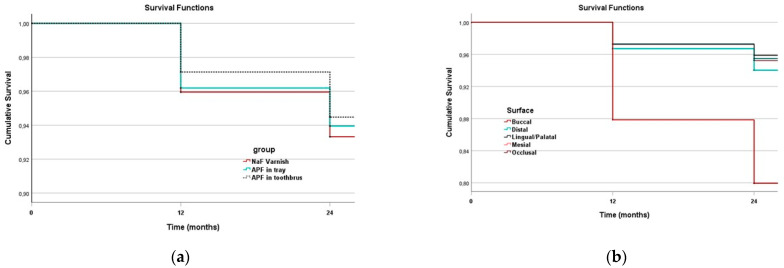
Survival analysis: (**a**) dental surfaces by group; (**b**) by type of surface.

The Kaplan–Meier survival curves underline statistically significant differences (Breslow estimator *p* = 0.03) between the APF in toothbrush group with respect to the NaF varnish and APF in trays groups (Figure 2a). The different tooth surfaces were analysed according to their type (Buccal, Distal, Lingual/Palatal, Mesial, and Occlusal). The occlusal surfaces (Figure 2b) had the lowest cumulative survival according to the Kaplan–Meier (Breslow estimator *p* < 0.01).

No gender or age difference was observed between groups.

The multivariate analysis (Cox-Regression) underlines a significant reduction in the risk rate of caries change in the APF in the toothbrush group; the status was highlighted (HR = 0.51 95%CI = 0.33/0.81). Sex and gender were not statistically significantly associated to caries increment. Table 4 displays the modification of the treatment needs between the three groups at baseline evaluation, at the 12th-month evaluation, and at the 24th-month evaluation; the modifications were not statistically significant for any group, but the trend analysis was statistically significant in the 24th-month evaluation (Cuzick’s test z = −2.21 *p* = 0.02).

## 4. Discussion

In this study, the use of an alternative application technique for the application of (APF) has been shown to be effective in maintaining healthy surfaces over 24 months.

Different caries preventive program strategies were carried out in many countries to decrease caries prevalence and incidence [16,17,18]. The current model of minimally invasive dentistry has suggested several models that prevent the loss of dental structure. The use of fluorides, especially the regular use of water and dentifrices, are the strategies with the most evidence in the control and prevention of dental caries [18,19]. The topical use of fluoride products in high concentrations (>2500 ppm) creates fluoride reservoirs, providing fluoride to the dental surface and promoting its penetration into the biofilm, being effective in reducing demineralization and increasing remineralization. This efficacy in caries prevention has been demonstrated in several in vitro and in situ clinical studies that pointed out that higher fluoride concentrations are needed to prevent this process [20,21,22].

Fluoride varnishes stand out in preventing dental caries, and are widely accepted by paediatric patients, especially for children under 6 years of age. Fluoride varnishes are widely used and indicated to control caries in children [1,21,23]. In the present study, this technique showed worse results compared to APF applied with a toothbrush or with tray techniques.

The fluoride gel/varnish applications are effective and the costs are offset in respect to restorative treatments taking into account that varnishes cost more but are more effective than gels [24,25]. Nevertheless, the costs of caries prevention are not achievable for the majority of countries worldwide, usually the only method for caries prevention available for the majority of children is through oral hygiene maintenance [3,26].

The present trial underlines that the use of APF on toothbrush provided better results than NaF varnish. This approach not only would be more economical than NaF varnish but also than the use of APF with trays.

Studies with supervised toothbrushing programs of up to 5 years show effectiveness especially in high-risk groups. In addition, a partnership with parents and oral care and nutrition counselling are essential to the success of the intervention [16].

A systematic review compared topical fluoride treatments with follow-up of at least one year and frequency of application. This study reaffirmed that the preventive effect of high fluoride gel on primary dentition has strong scientific evidence and is more effective when compared to permanent dentition [27,28].

Urquhart et al. [29] demonstrated that varnishes are the most effective preventive measure. In the present study, all three measures evaluated were effective when used as part of a school-based programme in controlling caries incidence.

As was expected, occlusal surfaces had worse survival than other dental surfaces. Evidence indicates that fissure sealants have been shown to be the most effective strategy in preventing occlusal caries in children and adolescents [29,30,31,32]. A limitation of this RCT study can be due to the brushing, as it was performed at home, without the possibility of a control by the authors of a regular adherence to the protocol and one time only at school, under the teachers’ supervision. However, this trial presents significant strengths: the long period of follow-up, the wide sample size and the evaluation of both primary and permanent teeth represent strengths that make the reliability of this study high. Fluoride compounds are still the most effective and affordable means of preventing caries, especially in countries where other preventive strategies at community level of certain efficacy, such as water fluoridation, are not implemented.

Some limits of the study design need to be underlined. First of all, the study population belonged to an age range, in which it was difficult to find a complete compliance and this might have affected the dropout. The number of subjects and the inclusion criteria do not allow us to generalize the results to the general population of this age group.

However, this trial presents significant strengths such as the long period of follow-up and the study design.

The main clinical significance of the trial is that the results might allow paediatric dentists and health authorities to select the new procedure as an active therapeutic agent to reduce dental caries.

## 5. Conclusions

The alternative technique of toothbrush application of APF gels has demonstrated better results in maintaining dental health for 24 months than NaF varnish and tray-applied APF, possibly due to the ability to better enter grooves and interproximal spaces. This technique is cost-effective and may offer advantages over traditional fluoride varnish application. A limitation of this study may be related to the capacity of the practitioner. Further studies should explore the efficacy and feasibility of self-applicable fluoride treatments in high-risk groups.

## Figures and Tables

**Table 1:** Caries Treatment Needs Index (CTNI).

CODE	Clinical Situation	Unit of Analysis	Treatment Plan
00	Sound teeth with history of preventive measures	Mouth	Preventive program: Low or moderate caries risk
01	Sound teeth without history of preventive measures		Preventive program: Low or moderate caries risk
02	Presence of initial caries lesions		Preventive program: High caries risk
03	Presence of cavitated lesions affecting enamel and/or dentine	1 quadrant	Preventive program: High caries risk + Restorative treatment
04		2 quadrants	Preventive program: High caries risk + Restorative treatment
05		3 quadrants	Preventive program: High caries risk + Restorative treatment
06		4 quadrants	Preventive program: High caries risk + Restorative treatment
07	Presence of cavitated lesions affecting enamel and/or dentine with pulp involvement	1 quadrant	Preventive program: High caries risk + Restorative treatment + Pulp treatment
08		2 quadrants	Preventive program: High caries risk + Restorative treatment + Pulp treatment
09		3 quadrants	Preventive program: High caries risk + Restorative treatment + Pulp treatment
10		4 quadrants	Preventive program: High caries risk + Restorative treatment + Pulp treatment
11	Presence of extensive cavitated lesions without possibilities of restorative treatment or presence of abscess or fistula	1 quadrant	Preventive program: High caries risk + Restorative treatment + Surgical treatment and eventual rehabilitation
12		2 quadrants	Preventive program: High caries risk + Restorative treatment + Surgical treatment and eventual rehabilitation
13		3 quadrants	Preventive program: High caries risk + Restorative treatment + Surgical treatment and eventual rehabilitation
14		4 quadrants	Preventive program: High caries risk + Restorative treatment + Surgical treatment and eventual rehabilitation

Calibration of the examiners and clinical examinations.

**Table 2:** Caries data (ICDAS) as the number of surfaces in groups for each ICDAS-merged categories recorded at baseline.

ICDAS Index	NaF Varnish *n* (%)	APF in Tray *n* (%)	APF in Toothbrush *n* (%)	*p*-Value
Sound surface (0)	5050 (90.60)	5167 (92.71)	5216 (93.63)	0.70
Initial caries (1–2)	39 (0.70)	33 (0.59)	28 (0.50)	0.82
Enamel caries (3)	61 (1.09)	67 (1.20)	61 (1.10)	0.86
Dentine caries (4–6)	424 (7.61)	306 (5.49)	266 (4.77)	0.68

**Table 3:** The changes of the CTNI approach from baseline to 12th and 24th months’ examinations between the three groups.

ICDAS Index	NaF Varnish *n* (%)	APF in Tray *n* (%)	APF in Toothbrush *n* (%)	*p*-Value
Sound surface (0)	5050 (90.60)	5167 (92.71)	5216 (93.63)	0.70
Initial caries (1–2)	39 (0.70)	33 (0.59)	28 (0.50)	0.82
Enamel caries (3)	61 (1.09)	67 (1.20)	61 (1.10)	0.86
Dentine caries (4–6)	424 (7.61)	306 (5.49)	266 (4.77)	0.68

Pearson’s χ^2^_(4)_ = 8.45 *p* = 0.08; Cuzick’s test for trend. Z = −1.52 *p* = 0.13.

**Table 4:** Modification of the treatment needs between the three groups at baseline evaluation, at the 12th-month evaluation, at the 24th-month evaluation.

CTNI Approach	NaF Varnish *n* (%)				APF in Tray *n* (%)				APF in Toothbrush *n* (%)		
	Baseline	12 ms	24 ms		Baseline	12 ms	24 ms		Baseline	12 ms	24 ms
*Pp*	20 (33.33)	16 (29.09)	14 (29.17)		16 (26.67)	12 (23.08)	11 (24.44)		30 (50.00)	30 (55.56)	27 (56.25)
*Pp + Rt*	25 (41.66)	29 (52.73)	30 (62.50)		30 (50.00)	34 (65.38)	30 (66.67)		17 (28.33)	17 (31.48)	16 (33.33)
*Pp + Rt + Pt*	15 (25.00)	10 (18.18)	4 (8.33)		14 (23.33)	6 (11.54)	4 (8.89)		13 (21.67)	7 (12.96)	5 (10.42)
	χ^2^_(4)_ = 6.66 *p* = 0.16				χ^2^_(4)_ = 5.96 *p* = 0.20				χ^2^_(4)_ = 2.98 *p* = 0.56		
**Baseline**				**12th Months Evaluation**				**24th Months Evaluation**			
χ^2^_(4)_ = 8.45 *p* = 0.07 Cuzick’s test z = −1.51 *p* = 0.13				χ^2^_(4)_ = 16.08 *p* < 0.01 Cuzick’s test z = −2.57 *p* = 0.01				χ^2^_(4)_ = 13.45 *p* < 0.01 Cuzick’s test z = −2.21 *p* = 0.02			

*Pp* = Preventive program; *Pp* + *Rt* = Preventive program + Restorative treatment; *Pp + Rt + Pt* = Preventive program+ Restorative treatment + Pulp treatment.

## Data Availability

The data presented in this study are available on request from the corresponding author.
